# The relationship between stage 1 and 2 non-small cell lung cancer and lung function in men and women

**DOI:** 10.1186/1471-2466-6-2

**Published:** 2006-02-01

**Authors:** Samir Malhotra, Stephen Lam, SF Paul Man, Wen Q Gan, Don D Sin

**Affiliations:** 1 Department of Medicine (Respiratory Division), University of British Columbia, All in Vancouver, British Columbia, Canada; 2 The James Hogg iCAPTURE Center for Cardiovascular and Pulmonary Research, St. Paul's Hospital, All in Vancouver, British Columbia, Canada; 3 British Columbia Cancer Agency, All in Vancouver, British Columbia, Canada

## Abstract

**Background:**

Reduced forced expiratory volume in one second (FEV_1_) has been linked to non small cell lung cancer (NSCLC). However, it is unclear whether all or only certain histological subtypes of NSCLC are associated with reduced FEV_1_. Moreover, there is little information on whether gender modifies this relationship. Using a large tissue registry, we sought to determine the relationship between FEV_1 _and subtypes of NSCLC and determine whether this relationship is modified by gender.

**Methods:**

We used data from patients who underwent tumor resection for NSCLC at a teaching hospital in Vancouver and had various pre-operative clinical measurements including FEV_1_. We divided the cohort into quartiles of predicted FEV_1 _and using both logistic and linear regression modeling techniques determined whether FEV_1 _was related to the occurrence of adeno or squamous cell carcinoma in men and women.

**Results:**

There were 610 patients in the study (36% females). On average, women were more likely to have adenocarcinoma than were men (72% of all cases of NSCLC in women versus 40% in men; p < 0.001). In women, there was no significant relationship between FEV_1 _and the risk of any histological subtypes of NSCLC. In men, however, there was an inverse relationship between the risk of adenocarcinoma and FEV_1 _such that the lowest quartile of FEV_1 _was 47% less likely to have adenocarcinoma compared with the highest FEV_1 _quartile (adjusted odds ratio, 0.52; 0.28 to 0.98; p for trend, 0.028). The reverse was observed for squamous cell carcinoma.

**Conclusion:**

In individuals undergoing lung resection for NSCLC, the risk of adenocarcinoma and squamous cell carcinoma of the lung varies as a function of FEV_1_, independent of smoking intensity in men but not in women.

**Clinical Implications:**

These data indicate that women are much more susceptible to adenocarcinoma than are men especially when they have normal or near normal lung function. It may thus be useful to conduct periodic surveillance chest radiographs in asymptomatic female smokers (or ex-smokers) to ascertain peripheral nodules or masses before distant metastases occur since adenocarcinomas tend to metastasize earlier in the disease course than squamous cell carcinomas.

## Background

Lung cancer is now an epidemic in women. Although the incidence of lung cancer has stabilized in men, there continues to be a dramatic increase in lung cancer cases in the female population. In 2003, an estimated 80,100 women in the United States (US) were diagnosed with lung cancer and 68,800 died from this disease [1]. In women, lung cancer has surpassed breast cancer as the leading cause of cancer mortality in 1987 and now accounts for 25% of all cancer deaths [1]. In both men and women, cigarette smoking is the leading causative factor for lung cancer. Compared to never-smokers, smokers have a 20-fold increase in the risk for lung cancer [2].

Interestingly, reduced forced expiratory volume in one second (FEV_1_), an important indicator of lung function, has also been linked with lung cancer, independent of the effects of cigarette smoking [3-5]. A study by Wasswa-Kintu et al. [6] found that FEV_1_, in a severity-dependent manner, was associated with lung cancer incidence and mortality even after adjustments for smoking intensity and status. Importantly, they found that even a very modest reduction in FEV_1 _(within the "normal" range of values) significantly increased the risk of lung cancer among current and former female smokers. In men, however, the increase in risk was much less pronounced. This study, however, did not include information on histological subtypes of lung cancer. It is therefore unclear whether the modifying effects of gender on the relationship between FEV_1 _and lung cancer impacted the risk for adenocarcinoma, squamous cell carcinoma or both. Such information would be valuable in gaining a better understanding of the potential differences (or similarities) in lung cancer susceptibility between men and women. To investigate this question, we analyzed data from a large cohort of individuals who underwent surgical resection for a small peripheral tumor.

## Methods

### Study participants

Data were obtained from patients who required surgical treatment of Stage 1 and II lung cancers at St. Paul's Hospital in Vancouver, Canada between 1978 and 2003. All resected tumors were examined by board-certified pathologists, who were unaware of the patient's lung function measurements at the time of the histological assessment. The histological subtype was diagnosed on surgical specimens according to the World Health Organization classification [7]. Because very few patients with small cell carcinomas undergo surgical resection, we restricted the analysis to non-small cell lung cancers (NSCLC). For analytic purposes, bronchoalveolar carcinoma was included in the adenocarcinoma group.

### Clinical variables

As part of their pre-operative work-up, all patients completed a pulmonary function test that met the standards of the American Thoracic Society [8]. From this test, FEV_1_, forced vital capacity (FVC) and transfer coefficient for carbon monoxide (K_CO_) were determined. We applied a prediction equation to calculate predicted FEV_1 _values for each patient [9]. Clinical information including age, gender, height, weight, and tobacco smoke exposure were also collected pre-operatively. Tobacco smoke exposure was estimated by using current smoking status, and the number of cigarettes smoked per day (pack-years of smoking). Arterial blood gas measurements on room air were also obtained in these patients.

### Statistical analysis

**Figure 1:**
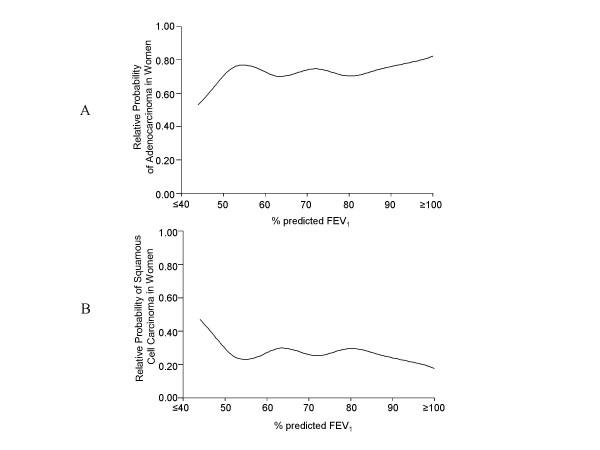
The Relative Risk Of Adeno (A) And Squamous Cell Carcinoma (B) According To Baseline FEV_1 _Measurement In Women Who Underwent Tumor Resection For Non-Small Cell Lung Cancer. Curves were fitted using a spline technique.

**Figure 2:**
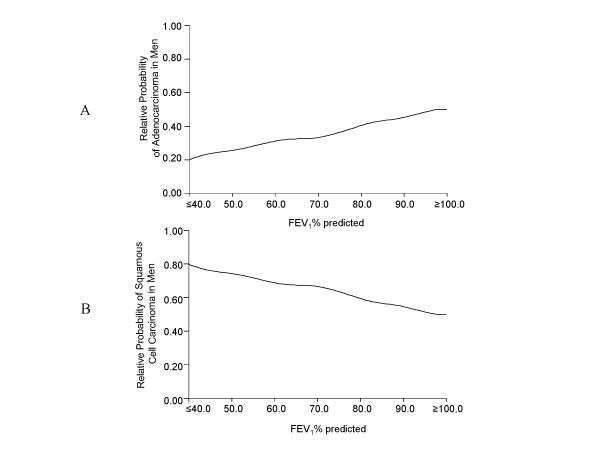
The Relative Risk Of Adeno (A) And Squamous Cell Carcinoma (B) According To Baseline FEV_1 _Measurement In Men Who Underwent Tumor Resection For Non-Small Cell Lung Cancer. Curves were fitted using a spline technique.

Continuous variables were compared using a Student t-test and dichotomous variables were compared using a chi-square test. To determine the relationship between FEV_1 _and the risk of adeno and squamous cell carcinomas in both men and women, we divided the groups into quartiles based on FEV_1 _within each gender category. We arbitrarily defined quartile 1 as the category with the highest predicted FEV_1 _and quartile 4 as the category with the lowest predicted FEV_1_. Using quartile 1 as the referent, we compared the odds of adenocarcinoma (or squamous cell carcinoma) across the quartiles of FEV_1 _in a logistic regression model. To the crude model, we added age, body mass index (BMI) and pack-years of smoking as covariates as they were significantly related to FEV_1 _and the risk of adenocarcinoma (and squamous cell carcinoma). These variables were included as both categorical and continuous variables. As there was no material difference in the results, for simplicity, we included them as continuous variables in the final model. To test for a linear increase (or decrease) in the odds ratios (OR) across the quartiles of FEV_1_, we used a chi-square test for trend, which adjusted for age, BMI and pack-years of smoking. We also performed a linear regression analysis in which we considered predicted FEV_1 _as a continuous variable rather than as quartiles to test the robustness of the data. We constructed fitted curves using a cubic spline method to visualize the results (figures 1 and 2). Similar analyses were performed using squamous cell carcinomas as the dependent variable. All analyses were conducted using SAS software version 9.1 (SAS Institute, Carey, N.C.) and SPSS version 11.0 (Chicago, IL). Two-tailed P-values less than 0.05 were considered significant. Continuous variables are expressed as mean ± SD, unless otherwise specified.

## Results

In total, there were 640 patients during the study period, who were diagnosed with NSCLC and who provided informed consent to use their lung tissue for research purposes. Twenty-two patients (3% of total) were excluded because they had undifferentiated tumors that could not be confidently classified into one of the two major histological subtypes of NSCLC. Additionally, eight patients were excluded due to insufficient information on the clinical variables of interest (e.g. FEV_1_). This left 610 patients for analysis.

The baseline characteristics of these patients are summarized in Table 1. There was a significant difference in the gender distribution of adenocarcinoma (40% in men vs. 73% in women; p < 0.001) and squamous cell carcinomas (57% in men vs. 24% in women; p < 0.001). Smoking status at the time of surgery was similar between male and female patients (p = 0.082), though overall, men had higher pack years of smoking compared with women (p < 0.001). Similarly, there was no difference in age between men and women (p = 0.496). Male patients had slightly lower FEV_1_, FVC and arterial oxygen tension (P_a_O_2_) than did female patients (Table 1).

Table 2 summarizes the unadjusted and adjusted odds ratios (controlling for covariates which included age, BMI and cigarette smoking) for adenocarcinoma and squamous cell carcinoma in women across quartiles of FEV_1_. In women, at all levels of FEV_1_, the odds of adenocarcinoma were much higher than that of squamous cell carcinoma (figure 1a and 1b). However, the odds did not vary as a function of FEV_1_.

In contrast, in men, the histological subtype varied significantly across the FEV_1 _quartiles (Table 3). Reduced FEV_1 _was associated with higher odds for squamous cell carcinoma, while at higher FEV_1 _values, adenocarcinomas predominated. These data did not change significantly when FEV_1 _was evaluated as a continuous rather than as a categorical variable (Figures 2a and 2b). Neither forced vital capacity nor transfer coefficient for carbon monoxide was significantly related to NSCLC histological subtypes in either men or women.

## Discussion

The most important and novel finding of the present study was that in women the risk for adenocarcinoma did not vary as a function of FEV_1_. At all levels of FEV_1_, adenocarcinomas predominated, constituting ~70% of all NSCLCs in the female population. In men, however, FEV_1 _made a material difference to the risk for adeno and squamous cell carcinomas. At reduced FEV_1_, men were far more likely to develop squamous cell carcinoma than adenocarcinomas; the reverse was true when FEV_1 _was in the normal or near normal range.

These results are consistent with those reported by Papi et al. [10] who found that individuals with chronic obstructive pulmonary disease (COPD) with mildly impaired FEV_1 _(~70% of predicted) had a four-fold increase in the risk for squamous cell carcinoma compared to those with normal lung function (FEV_1_, ~93% of predicted). However, this study could not comment on the potential modifying effects of gender because they had only 18 female subjects (representing 13% of the total subjects). We extend their findings by demonstrating that the risk of squamous cell carcinoma increases with decreasing FEV_1 _in men but not in women.

The mechanism(s) responsible for the apparent increase in the risk of adenocarcinoma in women are largely unknown. However, several possibilities exist. Firstly, there may be differences in genetic susceptibility. For instance, women for unknown reasons have more frequent mutations and transversions in the p53 gene than do men and aberrations in this gene have been associated with increased risk of adenocarcinoma [11]. Secondly, hormonal differences may impart differential risk of lung cancer between men and women. NSCLCs have been associated with increased expression of estrogen receptor β in chromosome 14 [12]. Stimulation of this receptor may have a role in the up-regulation of CYP1A1 gene. This latter gene encodes for an enzyme that has an integral role in the production of polycyclic aromatic hydrocarbons (Phase 1 enzymes), which may be carcinogenic [13]. Consistent with this notion, female smokers have increased expression of CYP1A1 than do male smokers [14]. Further investigation is required to investigate if estrogen has a causal link to adenocarcinoma by promoting carcinogenesis through increased CYP1A1 or other related pathways.

Thirdly, inflammation may play a primary role in the increased susceptibility of women to adenocarcinoma. Inflammation has been incited as an important part of the pathogenesis of certain types of lung cancer. Ardies [15] suggests that infection or toxic chemicals can incite a vigorous inflammatory reaction in the airways, producing certain by-products that may promote carcinogenesis. In NSCLC, cyclo-oxygenase 2 (COX-2) gene is over-expressed in nearly all stages of disease progression [16] and downstream enzymes of the COX-2 pathway involved in prostanoid synthesis such as prostaglandin E2 synthase is also up-regulated [17]. This inflammatory pathway has been implicated in the progression of lung cancer by inhibiting apoptosis, promoting angiogenesis and facilitating microinvasion of tumor to adjacent tissue, leading to tumor growth and metastasis [18,19]. Moreover, COX-2 activity has been associated with poorer prognosis in NSCLC [20]. Interestingly, Szczeklik and colleagues showed that in the asthmatic population, women were five times more likely to have a polymorphism in the COX-2 promoter region, -765G>C, compared to men (5% versus 1% frequency). This polymorphism is associated with increased production of prostaglandin (PG) E2 and D2 [21]. Indeed, monocytes cultivated from individuals with the C-C genotype produce levels of PGE2 and PGD2 that are 25 to 34 fold higher than that obtained from individuals with the G-G genotype [22]. The importance of this promoter polymorphism in the genesis of NSCLC is unknown.

Gender differences in the incidence of subtypes of NSCLC may also reflect subtle yet important differences in airway biology. In an experiment by Van Winkle et al. [23], female mice exposed to naphthalene, a chemical found in mainstream and sidestream smoke, were more likely to suffer airway damage than were male mice. Interestingly, for largely unclear reasons, the naphthalene-induced lung damage occurred predominantly in the distal airways, which is the usual site of adeno but not squamous cell carcinomas.

Adenocarcinomas tend be more peripherally located than are squamous cell carcinomas [2]. As such, patients are often asymptomatic until distant metastases occur. Unfortunately, by then, curative resection is not feasible. Our data indicate that women are much more susceptible to adenocarcinoma than are men especially when they have normal or near normal lung function. It may thus be useful to conduct periodic surveillance chest radiographs in asymptomatic female smokers (or ex-smokers) to ascertain peripheral nodules or masses before distant metastases occur. A large clinical study is needed to validate this notion and to determine the cost-effectiveness of this approach.

There are several limitations to the current study. Firstly, because individuals with severely impaired FEV_1 _do not usually undergo surgical resection for their lung cancer, the relationship between FEV_1 _and various cell types of lung cancer in individuals with FEV_1 _less than 50% of predicted remains unknown. Secondly, we did not have data for individuals with advanced stage lung cancers as these patients usually do not undergo surgery for their disease. However, there is no material reason to believe that inclusion or exclusion of such patients would change the findings of this study. Thirdly, we did not have data on small cell carcinoma because this type of lung cancer metastasizes very early on in the disease course and as such is not usually amenable to surgical resection. The effect of gender on the relationship between small cell carcinoma and FEV_1 _remains unknown.

In summary, the present study demonstrates that the risk of adeno- and squamous cell carcinoma of the lung varies as a function of FEV_1_, independent of smoking intensity in men but not in women. In women undergoing lung resection for cancer, adenocarcinoma predominates across all levels of FEV_1_. These data raise the possibility that there are fundamental differences in the airway biology between women and men, which confer increased susceptibility of adenocarcinoma for female smokers. Given that the incidence of lung cancers continue to rise in women, it is imperative to further investigate these gender differences such as the role of inflammation as well as hormonal, molecular and the genetic factors to explain why women are more prone to develop adenocarcinoma.

## Competing interests

The author(s) declare that they have no competing interests.

## Authors' contributions

SM and DDS participated in data acquisition, performed statistical analysis and drafted the manuscript. WG participated in data analysis and drafting of manuscript. SL provided important intellectual input to the analysis and in drafting of the manuscript. SFP participated in data interpretation, analysis and drafting of the manuscript. All authors read and approved the final manuscript.

## Pre-publication history

The pre-publication history for this paper can be accessed here:



## Figures and Tables

**Table 1:** A Comparison of Baseline Characteristics Between Men and Women With Non-Small Cell Lung Cancer, Who Underwent SurgeryContinuous variables are expressed as mean ± SD and dichotomous variables are expressed as % of column totals.

	Men N = 388	Women N= 222	P value
Age (years)	64 ± 9	64 ± 10	0.432
BMI (kg/m^2^)	26 ± 4	24 ± 5	<0.001
P_a_CO_2 _(mm Hg)	41 ± 8	40 ± 7	<0.001
P_a_O_2 _(mm Hg)	80 ± 22	86 ± 30	<0.001
Pre-bronchodilator FEV_1_(% predicted)	74 ± 19	85 ± 20	<0.001
Post-bronchodilator FEV_1 _(% predicted)	77 ± 18	88 ± 20	<0.001
Pre-bronchodilator FVC(% predicted)	86 ± 16	94 ± 18	<0.001
Post-bronchodilator FVC, (% predicted)	90 ± 15	96 ± 18	<0.001
K_CO _(% predicted)	78 ± 23	75 ± 22	<0.001
Current Smokers (%)	57	64	0.082
Pack Years of Smoking*	45 ± 2	37 ± 2	<0.001
Adenocarcinoma (%)	40	73	<0.001
Squamous cell carcinoma (%)	57	24	<0.001
Size of the tumors (cm)	3.6 ± 1.3	2.7 ± 1.0	0.008
Upper Lobe (%)	56	58	0.153
Middle Lobe or Lingula (%)	3	7	0.153
Lower lobe (%)	28	29	0.153
Multiple lobes (%)	5	3	0.153

* Expressed as geometric mean ± standard deviation

Abbreviations: BMI, body mass index, P_a_CO_2_, arterial carbon dioxide tension; P_a_O_2_, arterial oxygen tension, FEV1, forced expiratory volume in one second, FVC, forced vital capacity; K_CO_, transfer coefficient for carbon monoxide

**Table 2:** The Relative Odds for Adeno and Squamous Cell Carcinoma In Women Across Different Categories of Lung FunctionThe cells contain odds ratio (OR) and the 95% confidence intervals

Quartile of FEV_1_	1	2	3	4	Trend Test (p value)†
Range of FEV_1 _(% predicted)	103 to 149	88 to 102	74 to 88	43 to 74	
Number of subjects	55	56	55	56	
Unadjusted Odds for Adenocarcinoma	1 (reference)	0.83 (0.33–2.05)	0.35 (0.14–0.81)	0.75 (0.31–1.84)	0.216
Adjusted Odds for Adenocarcinoma*	1 (reference)	0.94 (0.36–2.48)	0.44 (0.18–1.09)	0.88 (0.34–2.32)	0.448
	1 (reference)				
Unadjusted Odds for Squamous Cell Carcinoma	1 (reference)	0.86 (0.32–2.32)	3.00 (1.25–7.18)	1.23 (0.48–3.13)	0.194
	1 (reference)				
Adjusted Odds for Squamous Cell Carcinoma*	1 (reference)	0.71 (0.25–2.06)	2.19 (0.85–5.61)	0.92 (0.33–2.54)	0.567

*Adjusted for age, body mass index and pack-years

†p-value for linear trend of increasing (or decreasing) odds ratio from quartile 1 to quartile 4

**Table 3:** The Relative Odds for Adeno and Squamous Cell Carcinoma In Men Across Different Categories of Lung FunctionThe cells contain odds ratio (OR) and the 95% confidence intervals

Quartile of FEV_1_	1	2	3	4	Trend Test (p value)†
Range of FEV_1 _(% predicted)	89 to 120	78 to 89	64 to 78	27 to 64	
Number of Subjects	97	97	97	97	
Unadjusted Odds for Adenocarcinoma	1 (reference)	0.88 (0.50–1.55)	0.55 (0.31–1.00)	0.48 (0.27–0.86)	0.004
Adjusted Odds for Adenocarcinoma*	1 (reference)	0.78 (0.43–1.44)	0.59 (0.32–1.09)	0.52 (0.28–0.98)	0.028
Unadjusted Odds for Squamous Cell Carcinoma	1 (reference)	1.28 (0.73–2.25)	1.79 (1.01–3.17)	2.35 (1.31–4.20)	<0.001
Adjusted Odds for Squamous Cell Carcinoma*	1 (reference)	1.43 (0.78–2.63)	1.65 (0.90–3.03)	2.09 (1.12–3.90)	0.019

*Adjusted for BMI, age and pack-years

†p-value for linear trend of increasing (or decreasing) odds ratio from quartile 1 to quartile 4
